# Antibiotic Susceptibility of Bartonella Grown in Different Culture Conditions

**DOI:** 10.3390/pathogens10060718

**Published:** 2021-06-08

**Authors:** Shiva Kumar Goud Gadila, Monica E. Embers

**Affiliations:** Division of Immunology, Tulane National Primate Research Center, Tulane University Health Sciences, Covington, LA 70433, USA; sgadila@tulane.edu

**Keywords:** *Bartonella*, azithromycin, antibiotic susceptibility, azlocillin, intracellular, MIC, MBC

## Abstract

Bartonellosis is caused by a Gram-negative intracellular bacterium with a zoonotic transmission. The disease, caused by any of several genospecies of *Bartonella* can range from a benign, self-limited condition to a highly morbid and life-threatening illness. The current standard of care antibiotics are generally effective in acute infection; these include azithromycin or erythromycin, doxycycline, gentamicin, rifampin, and ciprofloxacin. However, treatment of chronic infection remains problematic. We tested six different antibiotics for their ability to stop the growth of *Bartonella* sp. in the standard insect media and in an enrichment media. All antibiotics (ceftriaxone, doxycycline, gentamycin, azithromycin, ampicillin, and azlocillin) had minimum inhibitory concentrations (MICs) below 0.5 µg/mL in the BAPGM enrichment media but were ineffective at inhibiting growth when the standard insect media was used. Azlocillin was the most potent, with a MIC of 0.01 µg/mL. When *Bartonella* was tested under intracellular growth conditions, none of the antibiotics were efficacious singly. However, growth inhibition was observed when azlocillin and azithromycin were combined. These studies illustrate the impact of growth medium and intracellular environment on antibiotic susceptibility testing and indicate that azlocillin combined with azithromycin may be an effective drug combination for the treatment of Bartonellosis.

## 1. Introduction

Bartonellosis is the clinical disease described as Cat Scratch Disease (*Bartonella henselae*), Trench Fever (*Bartonella quintana*), or Carrion’s disease (*Bartonella bacilliformis*). Among 13 different *Bartonella* species or subspecies that were recognized as causative agents of human diseases, these three species of *Bartonella* were reported to be responsible for the majority of clinical illnesses [1,2]. In humans, *Bartonella* infections have been associated with several clinical abnormalities that include arthralgia, arthritis, bacillary angiomatosis, endocarditis, myocarditis, cutaneous lesions, granulomatous hepatitis, neuroretinitis, peliosis hepatis, pulmonary nodules, uveitis, and vasoproliferative tumors [1,3,4]. A most recent study has shown a possible association between *Bartonella* infection and malignant melanoma [5]. Using confocal microscopy techniques, the study has shown co-localization of *B. henselae* with vascular endothelial growth factor C (VEGFC), a melanoma growth factor, in the skin biopsy tissues from the patients with melanoma [5]. Another study focused on patients with neuropsychiatric disorders who reported concurrent cutaneous lesions [6]. Of these patients, 29/33 had positive serology or PCR for Bartonella. Considering the wide range of known clinical manifestations from the *Bartonella* infections, there is no single treatment that effectively works against all *Bartonella*-associated diseases.

Treatment regimens against *Bartonella* infections are primarily based on case reports that have limited access to the number of patients. As a result, treatment is confined to the immunological outcome of the disease rather than focusing on infective species [7]. Hence, the treatment that worked on an immunocompetent patient might not work on a patient whose immune system is compromised. Currently, gentamicin in combination with doxycycline is considered the best treatment regimen for the confirmed human cases of Bartonella endocarditis, and ceftriaxone with gentamicin is recommended for infective endocarditis when *Bartonella* infection is suspected [7,8]. For the treatment of *Bartonella* endocarditis in dogs and cats, doxycycline in conjunction with amikacin is recommended [9]. Erythromycin is the first-line antibiotic therapy for the treatment of patients with bacillary angiomatosis [7,10,11]. Rifampicin or streptomycin can be used to treat verruga peruana and have been used to treat other forms of *Bartonellosis* [7,12,13,14]. Recent case reports presented hepatosplenic complications of cat-scratch disease (CSD) in immunocompetent individuals [15,16]. Oral azithromycin is a proposed approach for the treatment of hepatosplenic CSD [16].

To avoid bacterial drug resistance, prolonged treatment periods must be avoided. New antibiotic combinations that are bactericidal, alongside antibiotics that could effectively penetrate the cell lipid barriers, considering the intracellular nature of *Bartonella*, should be taken into account in the treatment of *Bartonellosis*. These antibiotics should achieve therapeutic drug concentrations within the cells for the effective killing of the bacteria. Since a vaccine is not available, antibiotics are currently the mainstay of treatment. 

Previous studies on antibiotic susceptibility have evaluated the efficacy of antibiotics against early phase or stationary phase *Bartonella* cultures [17,18,19]. Inhibition in the growth of *B. henselae* in vitro was shown when tested with macrolides, tetracyclines, and rifampicin either using the Etest methodology [17,19,20] or the agar dilution method [19,20]. Considering the good intracellular activity of these antibiotics, macrolides and tetracyclines were used as first-line antibiotics for the treatment of diseases caused by *B. henselae* [18]. Minocycline, a tetracycline, was shown to be effective at a concentration <0.016 μg/mL against 32 isolates of *B. henselae* that were tested by Etest susceptibility [18]. A recent study has demonstrated that rifampin, erythromycin, azithromycin, doxycycline, and ciprofloxacin antibiotics that are currently used to treat *Bartonellosis* showed very poor activity against stationary phase *B. henselae,* but were effective against growing bacterial culture [21]. However, a 6-day treatment with the antibiotic combinations azithromycin/ciprofloxacin, azithromycin/methylene blue, rifampin/ciprofloxacin, and rifampin/methylene blue was able to completely eradicate the growth of the bacteria in log and stationary phases [22].

To evaluate the influence of culture media on antibiotic susceptibility testing (AST), we have investigated three different liquid culture media and assessed the efficacy of six different antibiotics: gentamicin, azithromycin, doxycycline, ceftriaxone, ampicillin, and azlocillin—both in cell-free liquid culture medium and in a cell-based culture system. We also tested drug combinations against *B. henselae*. Our report is the first study that tested the bactericidal activity of antibiotics both in vitro and upon coculture of *B. henselae* and DH82, an adherent canine macrophage-like cell line [23] that is permissive to *Bartonella* spp.

## 2. Results

### 2.1. Comparison of MIC with Different Media

**Figure 1:**
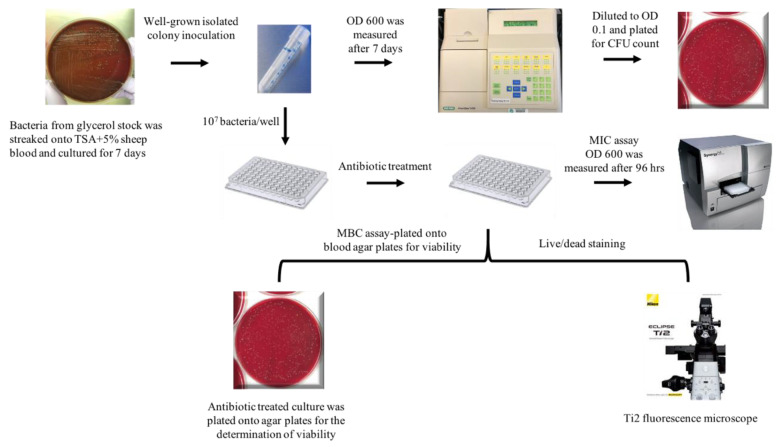
Experimental design for the evaluation of MIC and MBC values of antibiotics against *Bartonella* grown in different media.

Determination of susceptibility to antibiotics was performed in liquid culture media. Six different antibiotics were tested with multiple different concentrations against the pathogens *B. henselae* and *B. vinsonii*. The experimental design is shown in Figure 1. Ceftriaxone antibiotic dilutions (0.001, 0.01, 0.1, 0.3, 0.5, 1, 2, and 5 μg/mL), doxycycline dilutions (0.001, 0.01, 0.1, 0.3, 0.5, 1, 2, 5, and 10 μg/mL), gentamicin dilutions (0.01, 0.1, 0.3, 0.5, 1, 2, 4, 8, and 16 μg/mL), and azithromycin dilutions (0.001, 0.005, 0.02, 0.05, 0.1, 0.3, 0.5, 1, 2, 5, and 10 μg/mL), were tested based on previous antibiotic susceptibility studies [11,18,19]. 

In this study, we included azlocillin dilutions (0.001, 0.005, 0.01, 0.02, 0.5, 1, 2, and 5 μg/mL), and ampicillin dilutions (0.001, 0.005, 0.01, 0.02, 0.5, 1, 2, 4, and 8 μg/mL). Both the pathogens were susceptible to all the antibiotics that were used in BAPGM and Grace’s culture liquid mediums. MICs ranged from 0.01 to 0.1 μg/mL for ceftriaxone, doxycycline, and ampicillin, 2.0–4.0 μg/mL for gentamicin, 0.1–0.5 μg/mL for azithromycin, and 0.001–0.01 for azlocillin. Among all the antibiotics, azlocillin was more effective in inhibiting the growth of both the species of *Bartonella.* However, both of the bacterial species were not susceptible to any of the antibiotics when tested in Schneider’s insect medium (Table 1). 

### 2.2. Comparison of MBC

For this assay, both bacterial species, *B. henselae and B. vinsonii*, grown only in Grace’s insect media, were tested against all six antibiotics. While both were susceptible to all of the antibiotics, we found that doxycycline and ampicillin did not completely inhibit the growth of bacteria at concentrations 10 μg/mL and 8 μg/mL, respectively. However, ceftriaxone, gentamicin, azithromycin, and azlocillin were all bactericidal and completely eliminated the growth at concentrations below 10 μg/mL. The complete list of bactericidal concentrations of all the antibiotics is listed in Table 2.

**Figure 2:**
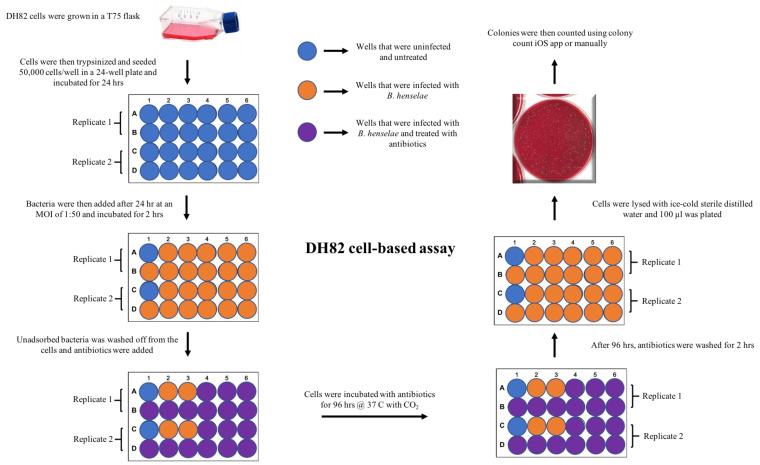
Experimental protocol for assessment of antibiotic efficacy against intracellular Bartonella.

**Figure 3:**
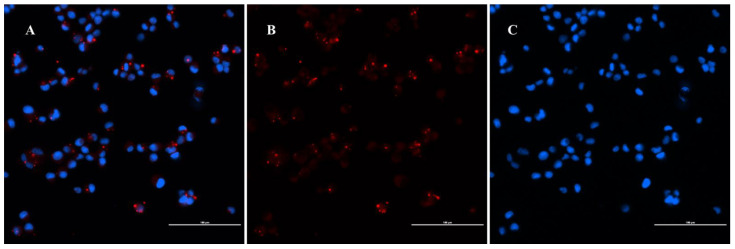
DH82 cells infected with *B. henselae*: Immunofluorescence staining of *B. henselae* was analyzed by using fluorescence microscopy. (**A**). merge image of *B. henselae* positive signal and DAPI. (**B**). red dots indicates positive staining for *B. henselae.* (**C**). DAPI staining cellular nucleus.

To evaluate the bactericidal activity and to determine if these antibiotics could penetrate the eukaryotic cell wall, we adapted a new DH82 cell-based coculture system (Figure 2). When *B. henselae* and DH82 cells were cocultured, we found that at an MOI of 50, about 83 ± 0.091% of DH82 cells were harboring intracellular *B. henselae* (Figure 3, Figure S2a,b). Cells that were uninfected with *B. henselae* were used as a negative control (Figure S1a). As an isotype control, DH82 cells infected with *B. henselae* and incubated with primary rabbit IgG were used (Figure S1b).

After confirming the rate of infectivity, we used the same MOI for testing antibiotics in this coculture system. When these antibiotics were used individually on the DH82 cell-based system, none of the antibiotics were potent against *B. henselae* at the concentration of 16 μg/mL (except for azithromycin and azlocillin) showed reduced bacterial growth but were unable to completely inhibit the growth (Table 2 and data not shown). Based on these data, we hypothesized that the antibiotic combinations of azithromycin/azlocillin would effectively inhibit the growth. Additionally, to determine β-lactam and macrolide combination efficacy, we used azithromycin/ampicillin combination for comparison. As hypothesized, the antibiotic combination of azithromycin/azlocillin was able to completely eliminate the growth of *B. henselae* at equal concentrations of <2 μg/mL. Surprisingly, the azithromycin/ampicillin combination was also effective at concentrations of 4 μg/mL. Similarly, we tested these combinations in the cell-free liquid culture medium. We observed similar results where the combination of azithromycin/azlocillin was more effective than the azithromycin/ampicillin combination (Table 3). The azithromycin/azlocillin combination was able to eliminate the growth of *B. henselae* at a concentration of 1 μg/mL, whereas the azithromycin/ampicillin combination was effective at 4 μg/mL of concentration.

### 2.3. Live/Dead Staining

**Figure 4:**
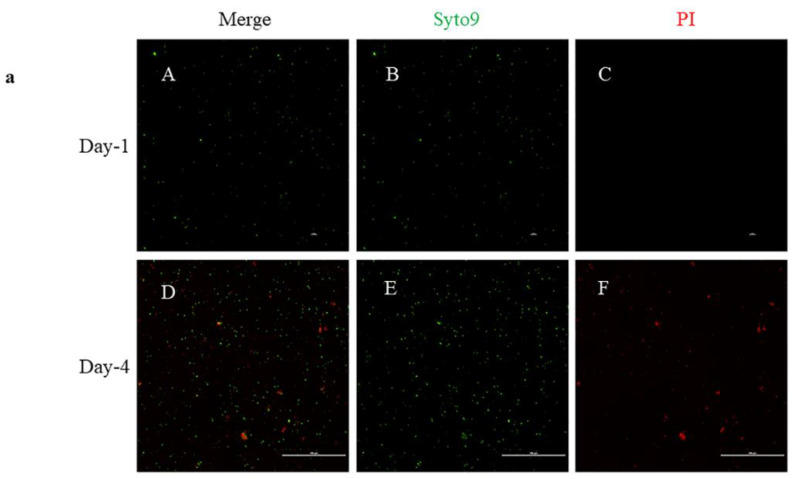

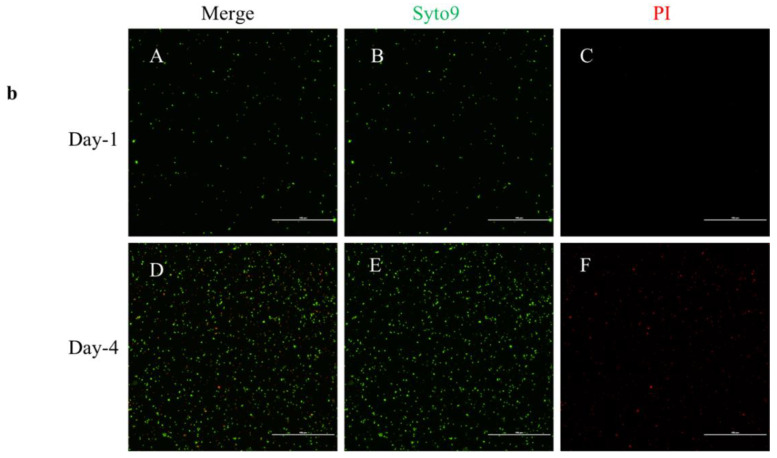
(**a**) Live/dead staining of *B. henselae*: *B. henselae* was stained on day-1 for viability before starting any assay. Table 1. Panel A indicates a composite image of both SYTO9 and PI staining, whereas the green signal indicates SYTO9 in panel B and red for PI in panel C. Similarly, the bottom panel D, E, F shows representative images of day-4. (**b**) Live/dead staining of *B. vinsonii*: Images shown are the representative images of *B. vinsonii* stained for viability before, upper panel A, B, and C and at the end of an assay, lower panel D, E, and F.

Bacteria were stained with the live/dead BacLight™ kit to determine the viability of the bacteria before every experiment. Both *B. henselae* and *B. vinsonii* were tested on the day of assay before antibiotic treatment, considering as time point 0 h. A viability test was also done after 96 h on control, untreated bacteria. Representative images of *B. henselae* are shown in Figure 3a and *B. vinsonii* in Figure 3b. On day-1 of the assay, the viable bacteria were around 97% compared to 73% after 96 h (Figure 4a,b). A decrease in the viable bacteria after 96 h could be due to the bacterial cells reaching the stationary phase. However, bacteria treated with antibiotics yielded inconclusive results. As such, there were bacteria that showed only SYTO9 signal when treated with azithromycin, ceftriaxone, and azlocillin concentrations of 10 μg/mL (Figure S3), the concentration at which there was no growth on blood agar plates. For this reason, live/dead staining was not taken into consideration in the determination of MBC either in cell-free liquid culture assay or DH82 cell-based assay

## 3. Discussion

We have tested the antibiotic susceptibility of *Bartonella* in both cell-based and cell-free liquid culture media systems. In AST, the primary role of the culture medium is to supply an optimal nutritional environment to support the growth of the test organism. In addition, the culture media should allow for the uniform distribution of antibiotics without any known or unknown chemical interactions with any media components [24]. Based on the results of susceptibility testing that were summarized in Table 1, bacteria grown in Schneider’s insect media showed high antibiotic resistance in the MIC assay. This resistance could be due to the chemical interaction of the antibiotics with the media components. Taking this into consideration, Schneider’s insect media was not used in the determination of MBC. Whereas, *Bartonella-Alphaproteobacteria* growth medium (BAPGM) [25] and Grace’s insect media showed none to minimal interaction with the media components. For this reason, *B. henselae* and *B. vinsonii* grown in Grace’s liquid media were used in this report for the MBC assay.

Both the *Bartonella* species were susceptible to all the antibiotics used in this study. The MICs of the antibiotics were <0.5 μg/mL, except for gentamicin, which is in accordance with the previous studies that tested for antibiotic susceptibility [18,20]. Higher MIC obtained for gentamicin is compatible with the results of testing other *B. henselae* isolates [18,26]. However, a most recent study has shown gentamicin MIC ranging in between 0.63–1.25 μg/mL when tested on stationary phase *B. henselae* [21]. The differences in these MIC values might be due to culture methods and the inoculum size. 

To more accurately determine the efficacy of these antibiotics, MBC assays were tested comparing DH82 cell-based and cell-free, liquid culture medium methods. Cell-free liquid culture media revealed that doxycycline, one of the drugs that are currently used in the treatment of *Bartonellosis* is bacteriostatic when used against *B. henselae* and *B. vinsonii* as there was growth on blood agar plates at the concentration of 10 μg/mL. Ampicillin was the other antibiotic that did not completely eliminate the growth of *Bartonella*. Ceftriaxone, gentamicin, azithromycin, and azlocillin were bactericidal at concentrations below 10 μg/mL, as these antibiotics completely eliminated the growth of bacteria on blood agar plates. However, these results were not consistent when tested on intracellular *Bartonella.* Doxycycline, gentamicin, ceftriaxone, and ampicillin had no effect on bacteria as there was a growth of *B. henselae* on agar plates at the concentration of 16 μg/mL. When used individually, azithromycin and azlocillin were the only drugs that showed a minimal effect in inhibiting growth. We hypothesized that the lack of penetration of these antibiotics through the DH82 cell line could be one of the reasons behind the insufficient efficacy of these antibiotics in inhibiting the *B. henselae* growth. Taking these results into consideration, we tested the combinations azithromycin/azlocillin and azithromycin/ampicillin, which resulted in complete inhibition of *B. henselae* growth. Since azithromycin is one of the first-line drugs recommended in the treatment of cat scratch disease [11] and also showed considerable efficacy in our single drug DH82 cell-based MBC assay, we evaluated the efficacy of only two two-drug combinations, which we found to be more active than single drugs alone. These drug combinations seem to be a more effective way to treat *Bartonella* infections, as a previous study also showed a complete elimination in the *B. henselae* growth when tested for drug combinations rather than exposure to single drugs [22].

Ampicillin, azlocillin, and ceftriaxone antibiotics belong to the antibiotics class of β-lactams. The primary targets of β-lactam agents are the penicillin-binding proteins (PBPs) on the bacterial cell wall made of peptidoglycan. These PBPs may interact with the beta-lactam ring of antibiotics that mimics the D-alanyl D-alanine portion of the peptide chain that is normally bound by PBPs. This interaction leads to the disruption of the peptidoglycan layer, leading to the lysis of the bacterium [27]. It has been reported that ceftriaxone is a recommended course of treatment in patients with *Bartonella* endocarditis [11,28]. According to our MBC assay on the coculture system, we speculate that ceftriaxone might be bactericidal when bacteria emerge outside of the erythrocytes, which is considered to be a reservoir site for *Bartonella* and also when found extracellularly. Previously, ampicillin was tested against 11 feline isolates of *B. henselae* by the Etest method to determine the MIC [26]. The lowest MIC of ampicillin that inhibited the growth of all the 11 isolates was <0.016 mg/L, which is consistent with our MIC data. Azlocillin is a β-lactam antibiotic that has recently garnered interest because it showed good efficacy against *Borrelia burgdorferi* both in vitro and in a mouse model [29]. Azlocillin was the most potent drug among all the antibiotics that were used in this study. Most importantly, the DH82 cell-based MBC antibiotic combination study revealed the efficacy of this drug in killing *B. henselae*. Here, the MBC of the azithromycin/azlocillin combination against intracellular *Bartonella* was similar to the cell-free liquid culture MBC concentration of azlocillin, when used alone. 

Aminoglycosides (AGs) inhibit protein synthesis by targeting either the 30 s or 50 s subunits of the bacterial ribosome. Notably, AGs interact with conserved sequences of 16 s rRNA of the 30 s subunit and cause misreading and premature termination of translations of mRNA. AGs enter into bacterial cytoplasm by an energy-dependent active bacterial transport mechanism, which requires oxygen and a proton motive force. For this reason, AGs work efficiently under aerobic conditions and have poor activity against anaerobic bacteria [30]. It was proposed that *Bartonella* can survive in microaerophilic environments by decreasing oxygen levels around the bacterium [31,32,33] and have an increased growth rate in the intracellular environment [34]. Taking this into consideration, we believe gentamicin alone might not be an efficient therapeutic treatment in the treatment of *Bartonellosis*. 

Similarly, tetracyclines interact with 16S rRNA of 30 s ribosomal subunit and prevent binding of t-RNA to the A site, whereas, macrolides bind to 23S rRNA of the 50 s subunit and inhibit protein synthesis [27,35,36]. Doxycycline, a tetracycline is currently used in combination with gentamicin, with a broad spectrum of activity in the treatment of *Bartonellosis*. Previously, an erythrocyte coculture model has shown that doxycycline, along with other β-lactam antibiotics were shown to not be bactericidal. The protein synthesis inhibitor antibiotics, tetracyclines, and macrolides were considered to have a synergistic effect when combined with antibiotics that can inhibit cell wall synthesis (such as beta-lactams), as it allows greater penetration of these antibiotics into the cell and requires lower doses [27]. Our drug combination data support this notion of the synergistic effect. 

Live/dead bacterial staining was inconclusive, yet there were a few interesting features when 7-day old liquid cultures of *B. henselae* were stained. There was a 60% reduction in the fluorescence signal intensity of SYTO9 in dead bacteria when compared to the live bacteria (Graph S-1). This result is not consistent with previous gram-negative bacterial staining, where SYTO9 fluorescence signal intensity was higher in dead bacteria compared to live bacteria [37]. However, the addition of PI to either live/dead bacteria did decrease the fluorescence signal intensity of SYTO9 (Supplementary figure, Graph-1) [37]. Based on our live/dead assay result, we conclude that the BacLight™ kit is more effective in distinguishing the live bacteria from the dead when *Bartonella* is in the exponential growth phase versus the stationary phase, at least when fluorescence microscopy is used. However, the fluorescence intensities for PI and SYTO9 were measured only once when the bacteria were stained in each experiment. 

Endothelial cells such as human umbilical vein endothelial cells provide an excellent in vitro model to study the pathogenesis of *Bartonella*, as there is evidence that endothelial cells are invaded in vivo [38,39,40]. Previous studies have also shown that *Bartonella spp*., invade and propagate in erythrocytes, suggesting that the intracellular location may protect the bacteria from antibiotics and the immune response [41,42]. Taking this notion into consideration, a study had tested the bactericidal activity of antibiotics against *B. quintana* and human erythrocytes by an in vitro coculture system [43]. In this study, we established a *Bartonella*-permissive cell line DH82 in testing antibiotic susceptibility, as an alternative to human endothelial cells. This cell line was used in previous studies for the isolation and PCR amplification of *Bartonella* sp., from the blood samples of dogs [44] and in *Bartonella* immunofluorescence serology testing [45,46,47].

A study that did a systematic review and meta-analysis of treatment outcome of human *Bartonellosis* has reported that the use of antibiotics gentamicin and doxycycline in the treatment of chronic bacteremia has improved the resolution rate significantly compared to no treatment. However, this treatment took a longer time to achieve cure compared to no treatment [13]. In the same study, it was reported that doxycycline did not show a statistically significant difference in the cure rate of bacillary angiomatosis in patients with HIV [48] compared to other treatments [13]. Based on our DH82 cell-based MBC data, we assume that the poor penetration rate of gentamicin into the cell [43] and the bacteriostatic nature of doxycycline could be the reason behind the outcome. Based on our combination drug study, we propose that the combination of azithromycin and azlocillin could be an effective regimen in the treatment of *Bartonellosis*. Our proposal of combination treatment is based on in vitro cell-based and cell-free based MBC assays. However, further studies are needed to evaluate the efficacy of these drugs in animal models. 

## 4. Materials and Methods

### 4.1. Bacterial Strains and Growth Conditions

The *B. henselae* strain used in this study is *San Antonio 2*, 267HO04, a human clinical isolate, and *B. vinsonii* subsp. *berkhoffii* TII, isolated from a sick dog. Both the bacteria were initially grown on tryptic soy agar supplemented with 5% defibrinated sheep blood plates that were either pre-made (Remel, R01198) or homemade, in a humidified atmosphere at 37 °C with 5% CO_2_ for seven days. For the preparation of liquid cultures, an individual well-grown colony from the plate was resuspended in 15 mL Falcon tubes containing 3 mL of either Schneider’s insect media supplemented with 10% FBS, or Grace’s insect media (Gibco, 11605-094) supplemented with 10% FBS, or BAPGM serum-free liquid media. The cultures grew for seven more days in the atmosphere as above without shaking. The number of viable bacteria was determined by either plating serial dilutions of the culture on plates for CFU or by live/dead staining. An illustration of the experiment is shown in Figure 1. Before every experiment, bacteria were mixed well and passed through a 22-gauge needle to break large clumps of bacteria. 

### 4.2. Live/Dead Staining

To determine the viability of the bacteria grown in liquid culture media, the LIVE/DEAD *Bac*Light™ Bacterial viability kit for microscopy (Thermofisher, L7012) was used according to the manufacturer’s protocol. Briefly, 7-day old liquid culture media was used for the determination of viability. Initially, bacteria were diluted down to an OD of 0.1 using the respective liquid culture media. One milliliter of 0.1 OD bacterial culture was then transferred into a 2 mL microcentrifuge tube and centrifuged at 7000 rpm for 10 min. After removing the supernatant, the pellet was resuspended in 1 mL of 0.85% NaCl and the bacteria were pelleted again. After three washes, bacteria were resuspended in 1 mL of 0.85% NaCl and incubated with 1 μL of green-fluorescent nucleic acid dye (SYTO9) and 1μL of red-fluorescent nucleic acid dye (PI) for 15 min. All the incubations were done at room temperature and in the dark. After 15 min of incubation with both the dyes, bacteria were visualized under fluorescence microscopy and images were acquired using Nikon NIS elements software. Later, the mean fluorescence intensity of both the SYTO9 and PI was measured using the Nikon elements software. Mean fluorescence values were acquired from a total of 10 images per experimental condition. Alternatively, to obtain dead cells, bacteria were treated with 70% isopropanol and incubated for 1 h, while the sample was mixed for every 15 min and stained as above. Additionally, the entire staining protocol was repeated by staining the bacteria with PI first and then with SYTO9 both on dead and live bacteria and the fluorescence values were recorded accordingly. However, in the staining of antibiotics treated bacteria, both the dyes were pre-mixed into 1ml of sterile-filtered distilled water, as previously described [49] and 10 μL of the mixture was added into each well, incubated and visualized as described above. 

### 4.3. DH82 Cell Culture and Seeding Density

DH82 cells (obtained from the Breitschwerdt lab) were initially cultured in T75-Flasks (Corning, 430641U) until confluency and were split at a ratio of 1:4 for every 2–3 days. Cells were then seeded into either 24-well cell culture plates (Corning, 3526) for coculture assay or 8-well chamber slides (Millipore# PEGGS0816) for immunofluorescence assays. A total of 50,000 cells were seeded into each well of a 24-well plate or per chamber of an 8-well chamber slide with EMEM + 15% FBS and incubated for 24–48 h before infections. 

### 4.4. Minimum Inhibitory Concentration (MIC) and Minimum Bactericidal Concentration (MBC) Assays

On the day of assay, optical density (OD) of 7-day old bacterial liquid culture that was grown from an individual colony was measured using a spectrophotometer (Bio-rad, SmartSpec 300, 170-2501). Bacterial culture was then diluted down to an OD of 0.1 ≈ 3 × 10^8^ cfu/mL with the respective media. 

To determine the MIC of the antibiotics and for the visualization of the bacterial growth, a clear 96-well plate was used. Into each well of a 96-well plate, 50 μL of the 0.1 OD bacterial culture and 50 μL of the liquid media containing antibiotics were added to the wells, bringing the total volume to 100 μL of 0.05 OD bacteria (CFU ≈ 1.5 × 10^7^) in each well. Wells containing only bacteria with the solvent used in the dilution of the antibiotics served as a positive control to verify the growth of bacteria. As a negative control, wells containing only media and solvents were used. Each concentration of diluted antibiotic was run in four replicates to minimize the error rate. The 96-well plate was covered with the lid and incubated at 37 °C with 5% CO_2_. OD of the culture was read after 48 h and 96 h using a hybrid multi-mode microplate reader (Biotek, Synergy H4 hybrid reader) at OD_600_. The assay was repeated three times. 

For the MBC assay, the antibiotic concentrations that showed inhibition of bacterial growth with MIC assay were used. Briefly, the bacterial culture that was treated with antibiotics for 96 h was collected into 1.5 mL microcentrifuge tubes and centrifuged at 7000 rpm for 10 min. The supernatant was removed, and the bacterial pellet was washed with 1X PBS twice. The bacterial pellet was then resuspended in 100 μL of 1 × PBS, plated onto blood agar plates, and incubated at 37 °C with 5% CO_2_ for about 14 days. This assay was repeated three times with all six different antibiotics and different concentrations. 

### 4.5. Antibiotics and Their Dilution

Antibiotic stocks were prepared in the following solvents: 10 mg/mL of ceftriaxone (USP), doxycycline (Fisher scientific, D9891-1G), gentamicin (Fisher scientific, 1405-41-0), ampicillin (Fisher scientific, 69-52-3), and azlocillin (Flightpath) in sterile cell culture grade water, whereas 5 mg/mL of azithromycin (Sigma, PZ0007-5MG) was dissolved in sterile cell culture grade DMSO. Antibiotics were then aliquoted and stored at either −80 °C or −20 °C until further use. On the day of assay, these stock concentrations were diluted to 1 mg/mL and 100 μg/mL in the respected bacterial culture media for MIC and cell-free, liquid culture MBC assays. For the DH82 cell-based MBC assay, antibiotics were diluted in EMEM media supplemented with 15% heat-inactivated FBS. 

### 4.6. DH82 Cell-Based, MBC Assay

Bacteria (*B. henselae*) that were grown in Grace’s insect media, as previously described, were centrifuged at 7000 rpm for 10 min. The supernatant was removed and washed with 1X PBS three times. After the washes, the pellet was resuspended in EMEM media supplemented with 15% heat-inactivated FBS. This bacterial suspension was then added to DH82 cells at a multiplicity of infection (MOI) of 1:50 and incubated for 2 h. After 2 h, unbound bacteria were removed by washing the cells with only EMEM media three times each in 15 min washes. Cells were then incubated with EMEM + 15% FBS media at 37 °C with 5% CO_2_ for 48 h. 

After 48 h of incubation, the media was removed from the cells, and antibiotics that were diluted in EMEM + 15% FBS were added. Each antibiotic was diluted at 11 different concentrations (16, 8, 4, 2, 1, 0.5, 0.25, 0.125, 0.062, 0.031, 0.0156 μg/mL), as described above and incubated for 96 h. This incubation period was determined based on our cell-free, liquid culture MBC data. The bactericidal activity of the antibiotics was then evaluated by plating the lysed cells on blood agar plates. Briefly, infected DH82 cells were initially washed for 30 min twice to remove the antibiotics that were added. Later, cells were osmotically lysed by adding 1 mL of sterile ice-cold distilled water and incubated for 5 min on ice [50,51]. The lysed cells were then plated onto blood agar plates and incubated at 37 °C with 5% CO_2_ for about 10 days to evaluate the growth of viable *B. henselae*. This experiment was conducted in duplicates and repeated twice with all six antibiotics including 11 different concentrations. The experimental overview is presented in Figure 2. The untreated infected cells were used as a positive control and untreated uninfected cells served as a negative control. The CFU counts in this paper were presented as average ± standard deviation. 

### 4.7. Immunofluorescence Assay

After 48 h of infection with *B. henselae,* DH82 cells were subjected to immunofluorescence assays. This seeding of the cells and the infection was performed on 8-well chamber slides (Millipore# PEGGS0816). Initially, cells were fixed with chilled ethanol and acetic acid at a ratio of 2:1 and incubated at −20 °C for 10 min. Cells were washed with 1× PBS three times by gentle rocking. Next, cells were permeabilized using 0.1% Triton-X 100 dissolved in 1× PBS and incubated for 20 min. After 20 min, Triton-X was washed from the cells using 1× PBS. Cells were then covered with blocking buffer (10% Normal Goat Serum-Gibco, 16-210-072, in PBS) for one hour at room temperature. After blocking, cells were incubated with the primary antibody: rabbit polyclonal anti–*B. henselae* (serum derived from a hyperimmunized rabbit eight weeks after inoculation with in vitro propagated *B. henselae*) at a dilution of 1:300 for 1 h followed by three 1× PBS washes. Cells were then incubated with secondary antibody Goat Anti-rabbit IgG-Alexa Fluor 594 (Thermo Fisher Scientific, R37117) diluted 1:1000 in blocking buffer. Slides were washed after incubation with the primary antibody, then partially dried by tapping the glass edge onto a paper towel. Cells were counterstained with DAPI for 10 min to stain nuclei (EMD millipore, 2160) and mounted with the anti-quenching solution (Thermofisher, P36934) and coverslipped. To prevent the decay of fluorescent signal intensity, slides were examined and photographed within one week of completion of the staining procedure. As a negative control, uninfected cells were used. This experiment was repeated three times and the percentage of infection was reported as average ± standard deviation.

Imaging was performed using a Nikon Ti2-E motorized fluorescence microscope [52]. Infected and uninfected cells were imaged during the same session with identical acquisition parameters. Fluorescence intensity was optimized on uninfected cells to eliminate the autofluorescence from the cells and remained constant for all the infected cells. When made, adjustments to brightness, contrast, or color balance were applied to the whole image. 

## Figures and Tables

**Table 1:** Comparison of MIC values for different antibiotics with two strains of *Bartonella*.

*B. henselae*				*B. vinsonii*	
	MIC (µg/mL)			MIC (µg/mL)	
Antibiotics	Schneider’s	BAPGM	Grace’s	Grace’s	Antibiotics
Ceftriaxone	>0.3	0.01–0.1	0.01–0.1	0–0.01	Ceftriaxone
Doxycycline	>0.3	0.01–0.1	0.01–0.1	0.01–0.1	Doxycycline
Gentamicin	>1	0.1–0.5	2.0–4.0	2.0–4.0	Gentamicin
Azithromycin	>0.02	0.005–0.02	0.3–0.5	0.1–0.3	Azithromycin
Azlocillin	>2	0.01–0.02	0.005–0.01	0.001–0.005	Azlocillin
Ampicillin			0.02–0.1	0.01–0.02	Ampicillin

**Table 2:** Differences in the MBC of antibiotics tested against extracellular and intracellular Bartonella.

	Cell-Free, Liquid Culture Assay		DH82 Cell-Based Assay
	MBC (µg/mL)		MBC (µg/mL)
Antibiotics	*B. henselae*	*B. vinsonii*	*B. henselae*
Ceftriaxone	2.0–4.0	0.5–1.0	>16
Doxycycline	>10	>10	>16
Gentamycin	2.0–4.0	4.0–8.0	>16
Azithromycin	5.0–10	2.0-5.0	>16
Azlocillin	1.0–2.0	0.25–0.5	>16
Ampicillin	>8	>8	>16

**Table 3:** Efficacy of Two Antibiotic Combinations against Intracellular Bartonella.

		DH82 Cell-Based Assay	Cell-Free, Liquid Culture Assay
Antibiotic Combination	Concentration (µg/mL)	CFU/mL after 96 h of Drug Exposure	CFU/mL after 96 h of Drug Exposure
Drug free control	0	1.03 ± 0.76 × 10^4^	1 ± 0.1 × 10^9^
Azithromycin + ampicillin	16	0	0
	8	0	0
	4	0	3.5 ± 2.12
	2	10	37.5 ± 3.54
	1	ND	60.5 ± 6.36
	0.5	ND	1.25 ± 0.35 × 10^3^
Drug free control	0	0.6 3± 0.61 × 10^4^	1.15 ± 0.21 × 10^9^
Azithromycin + azlocillin	16	0	0
	8	0	0
	4	0	0
	2	0	0
	1	ND	11 ± 4.24
	0.5	ND	2.02 ± 0.33 × 10^2^

## Data Availability

All data are available within the manuscript and supplemental information.

## Supplementary Materials

The following are available online at https://www.mdpi.com/article/10.3390/pathogens10060718/s1, Figure S1: (a) Immunofluorescence staining of B. henselaein DH82 cells: representative images of uninfected control DH82 cells. DH82 cells were stained with primary rabbit B. henselaehyperimmunized polyclonal (primary) and goat anti-rabbit IgG Alexa fluor 594 (secondary) antibodies. (b) isotype control:DH82 cells that were infected with B. henselaeused as an isotype control. Cells were stained with primary Rabbit IgG isotype control (Thermofisher# 02-6102) and secondary goat anti-rabbit IgG Alexa fluor 594 secondary antibody. Scale bar is 10 μm. Image was acquired with a Nikon fluorescence microscope using a 60× objective. Figure S2: (a) 24-hr after infection:Infection rate of DH82 cells with B. henselaewas assessed. DH82 cells were infected with B. henselaeat different ratio of infections from 1:1 to 1:1000 and stained for B. henselaeusing IFA assay. (b) 48 hrsafter infection:number of DH82 positive cells were assessed after 48 hrsof infection with different MOIs from 1:1 to 1:1000. Images shown are the representative images of 24 hrsinfection and 48 hrsinfection. Figure S3: Live/dead staining of B. henselaetreated with antibiotics. Figure S4: PI and SYTO9 staining analyzed with fluorescence microscopy.
