# Supplementary material for: Antibiotic Susceptibility of Bartonella Grown in Different Culture Conditions
Source: Pathogens. 2021 Jun 8;10(6):718. doi: 10.3390/pathogens10060718 (PMC8229624; doi:10.3390/pathogens10060718)
Supplement: Supplementary file 1 [file pathogens-10-00718-s001.zip › pathogens-1250348-supplementary.pdf]

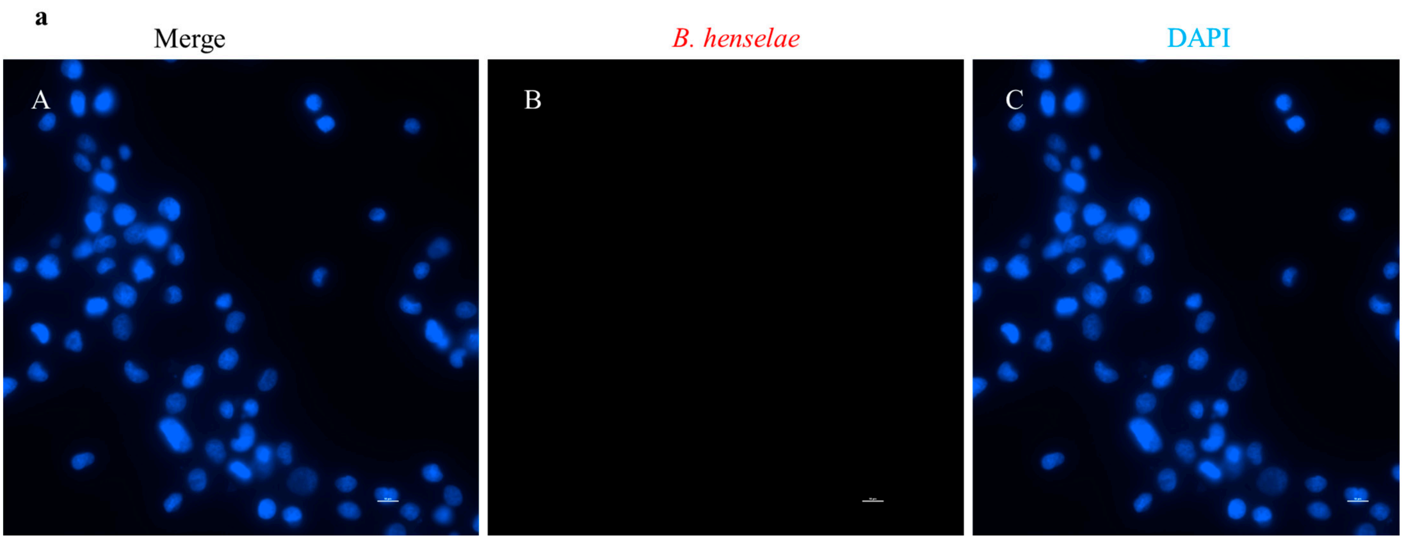

(**a**)

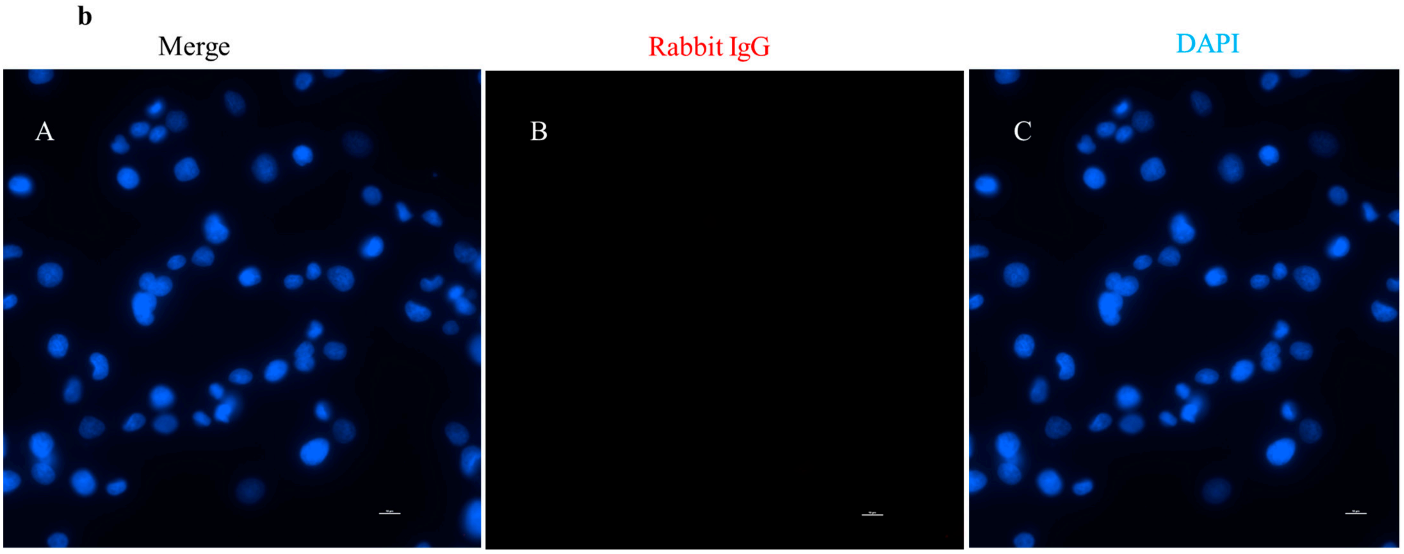

(**b**)

Figure S1 a) Immunofluorescence staining of *B. henselae* in DH82 cells: representative images of uninfected control DH82 cells. DH82 cells were stained with primary rabbit *B. henselae* hyperimmunized polyclonal (primary) and goat anti-rabbit IgG Alexa fluor 594 (secondary) antibodies. **b) isotype control:** DH82 cells that were infected with *B. henselae* used as an isotype control. Cells were stained with primary Rabbit IgG isotype control (Thermofisher# 02-6102) and secondary goat anti-rabbit IgG Alexa fluor 594 secondary antibody. Scale bar is 10µm. Image was acquired with a Nikon fluorescence microscope using a 60x objective.

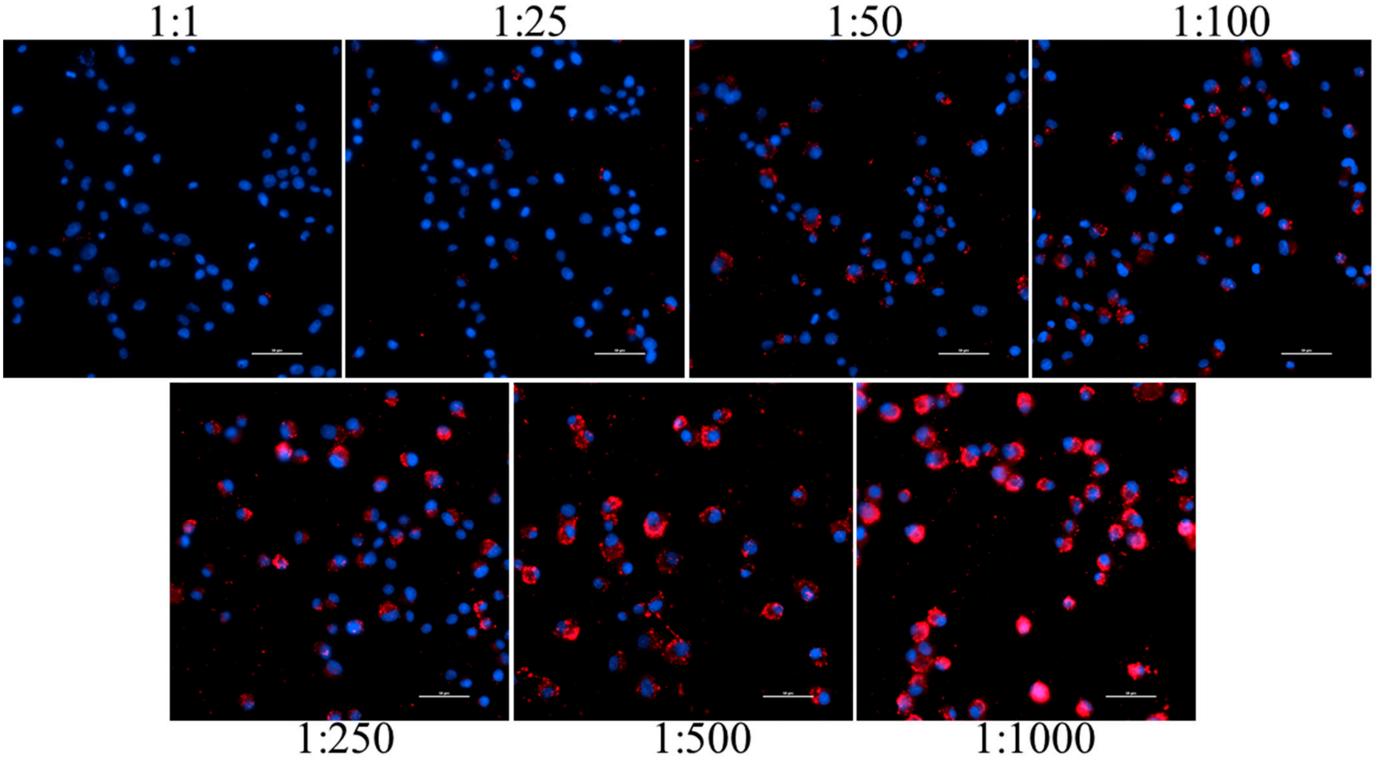

(**a**)

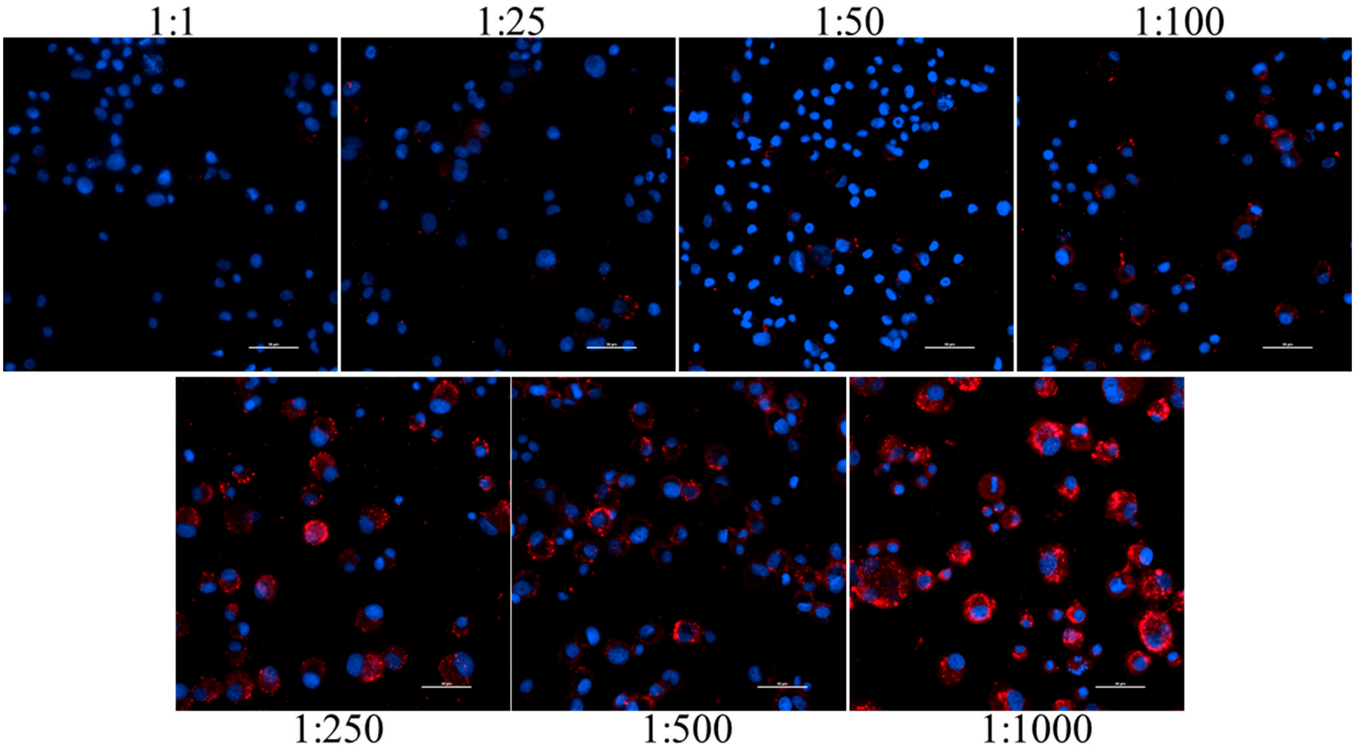

(**b**)

Figure S2 a) 24-hr after infection: Infection rate of DH82 cells with *B.* *henScheme 82.* cells were infected with *B. henselae* at different ratio of infections from 1:1 to 1:1000 and stained for *B. henselae* using IFA assay. **b) 48 hrs after infection:**

number of DH82 positive cells were assessed after 48 hrs of infection with different MOIs from 1:1 to 1:1000. Images shown are the representative images of 24 hrs infection and 48 hrs infection.

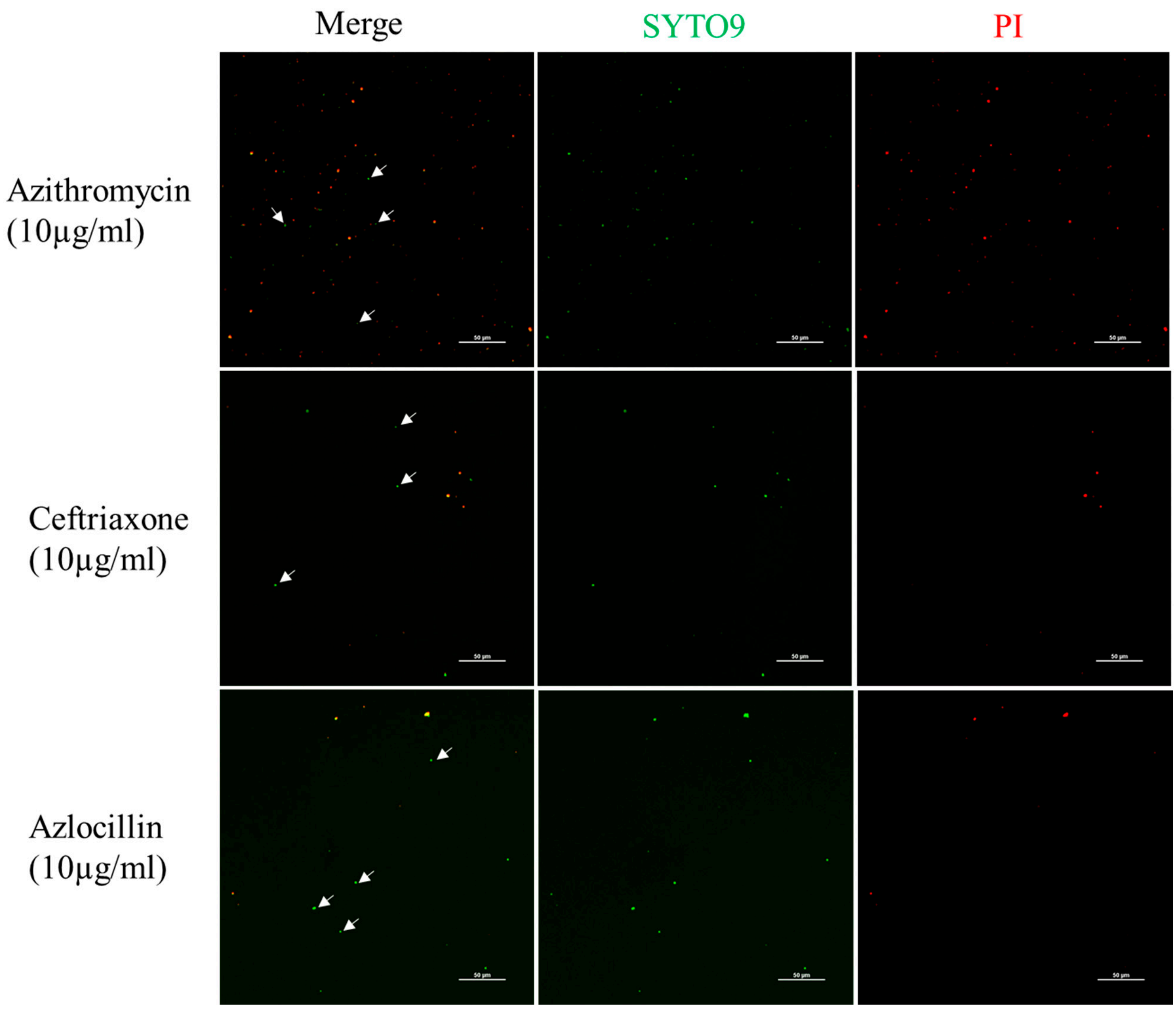

Figure 3. Live/dead staining of *B. henselae* treated with antibiotics: A) representative image of *B. henselae* after azithromycin treatment with a concentration of 10µg/ml. **B)** representative image of *B. henselae* after ceftriaxone treatment with a concentration of 10µg/ml and **C)** representative image of *B. henselae* post-treatment with azlocillin at a concentration of 10µg/ml. White arrows point to *B. henselae* that were stained for only SYTO9 indicating viability.

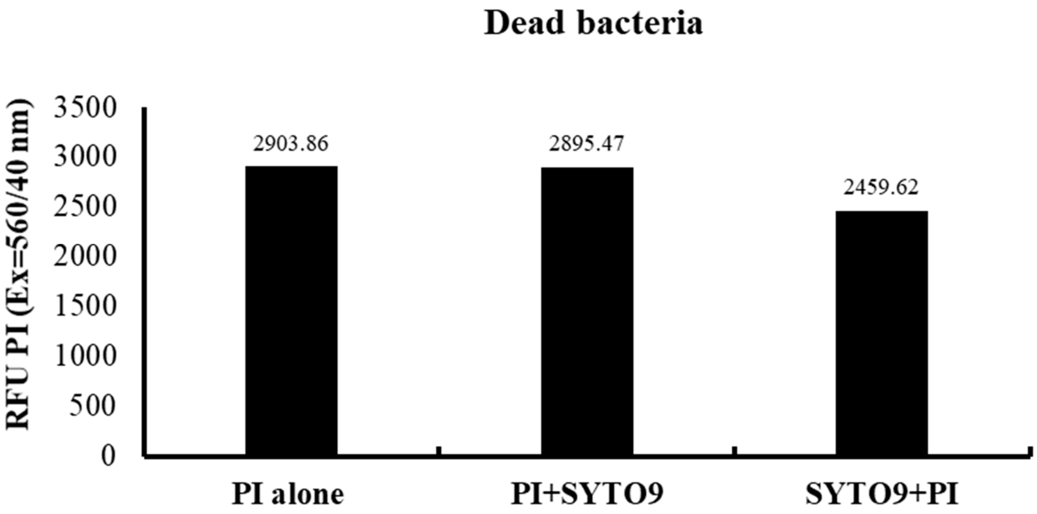
Dead bacteria
RFU PI (Ex=560/40 nm)
3500
3000
2500
2000
1500
1000
500
0
2903.86
2895.47
2459.62
PI alone
PI+SYTO9
SYTO9+PI

(A)

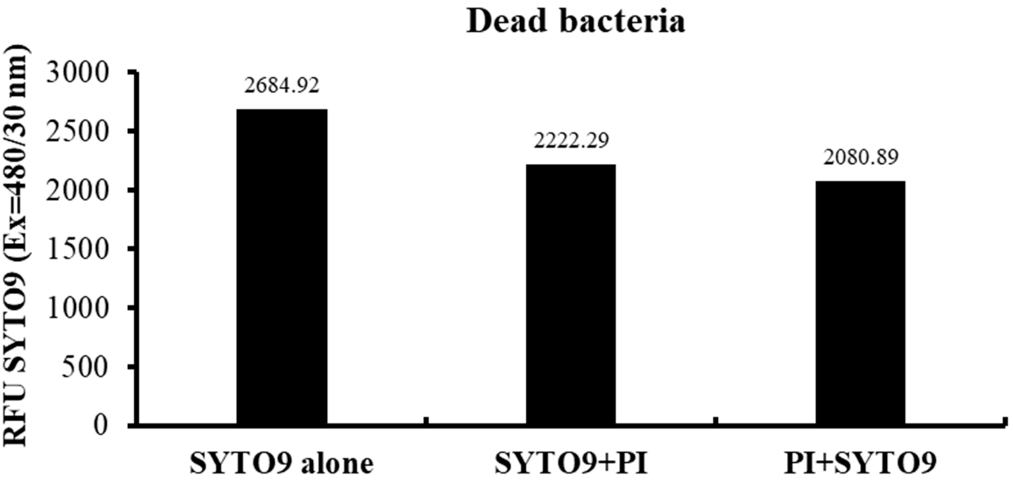
Dead bacteria
RFU SYTO9 (Ex=480/30 nm)
3000
2500
2000
1500
1000
500
0
2684.92
2222.29
2080.89
SYTO9 alone
SYTO9+PI
PI+SYTO9

(**B**)

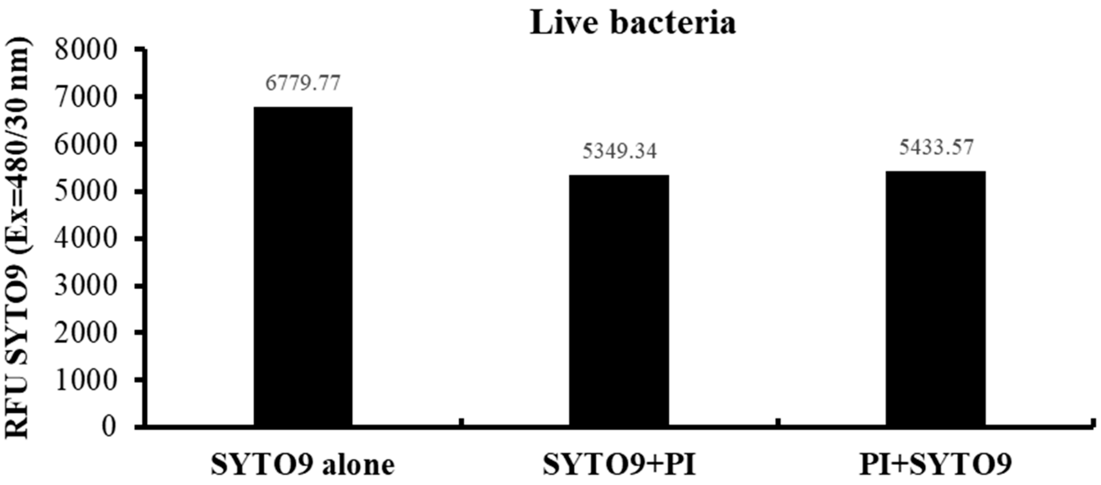
Live bacteria
RFU SYTO9 (Ex=480/30 nm)
8000
7000
6000
5000
4000
3000
2000
1000
0
6779.77
5349.34
5433.57
SYTO9 alone
SYTO9+PI
PI+SYTO9

(**C**)

Figure S-4: PI and SYTO9 staining analyzed with fluorescence microscopy: Relative fluorescence intensity (RFU) at 560/40 nm and 480/30 are shown for live and dead *B.* ***henselae*. To determine the RFU values, dead/live bacteria was initially stained with either SYTO9 or PI alone and fluorescence intensity was measured. In the next round, live/dead bacteria were stained with either PI or SYTO9 for 5 min and then after adding second stain bacteria were incubated for 15 more min. After 20 min, fluorescence intensity of both SYTO9 and PI were measured **A) shows RFU values of PI for dead bacteria **B)** shows RFU values of SYTO9 for dead bacteria **C**) shows RFU values of SYTO9 for live bacteria. Here, RFU values of PI for live bacteria were not shown as most of the bacteria were viable and PI was expressed in only 3% of the bacterial cells.
